# Development of a Scoring Method Based on a Chest CT Scan to Determine the Outcomes of COVID-19 Patients

**DOI:** 10.7759/cureus.47354

**Published:** 2023-10-19

**Authors:** Ali Bidari, Elham Zarei, Morteza Hassanzadeh, Milad Gholizadeh Mesgarha, Arash Pour Mohammad, Reyhaneh Shafiei, Mahsa Mortaja, Mahya Naderkhani

**Affiliations:** 1 Department of Rheumatology, Iran University of Medical Sciences, Tehran, IRN; 2 Department of Radiology, Iran University of Medical Sciences, Tehran, IRN; 3 Department of Internal Medicine, Iran University of Medical Sciences, Tehran, IRN; 4 School of Medicine, Iran University of Medical Sciences, Tehran, IRN; 5 Department of Dermatology, Iran University of Medical Sciences, Tehran, IRN; 6 Cancer Research Center, Tehran University of Medical Sciences, Tehran, IRN; 7 Emergency Medicine Management Research Center, Iran University of Medical Sciences, Tehran, IRN

**Keywords:** covid-19, prediction scoring system, chest ct scan, severity scoring, prognostic scores, mortality prediction, intubation, icu admission, death, sars-cov-2

## Abstract

Introduction

As COVID-19 shifts from pandemic urgency to endemic management, healthcare systems are faced with the evolving challenge of providing optimized care and adept resource allocation in this evolving landscape of the disease. However, the timely management and accurate assessment of disease severity remains a cornerstone of effective treatment. This study presents a pioneering scoring system, based on the primary chest CT scan findings, to predict patient outcomes and to equip clinicians with a tool that can expedite decision-making.

Method

A retrospective cohort study was conducted involving 406 confirmed COVID-19 cases referred to two of our hospitals in Tehran, between February and April 2020. Radiographic and CT scan data were sourced from the imaging archive system and evaluated by a certified radiologist. We devised distinct severity scores for CT findings, demographic factors, and clinical indicators. These were synthesized into a comprehensive severity score to forecast critical patient outcomes, such as mortality, ICU admission, intubation, or extended hospitalization. Of the total cases, 161 (39.7%) were classified as severe, while 245 (60%) fell into the low or moderate severity category.

Results

The mean score of demographic, CT scan, and clinical characteristics was significantly higher for those in the severe COVID-19 than the non-severe group. The cutoff score for predicting the outcomes in COVID-19 patients for demographic, clinical, and chest CT scan factors was 2.5, 9.5, and 8.5, respectively. Multivariate analysis indicated that each unit increase in these scores elevated the odds of fatal outcomes by 24%, 2.8%, and 12%, respectively. Then, using the comprehensive severity score, which is the sum of the above scores, we further predicted the disease severity.

Conclusion

The findings suggest that our innovative scoring system, based on initial chest CT scan findings, serves as a robust predictor of COVID-19 outcomes.

## Introduction

In late December 2019, Wuhan, China, became the epicenter for a surge of severe pneumonia cases, later attributed to the SARS-CoV-2 virus, leading to the identification of COVID-19 as a distinct disease. By February 2020, the World Health Organization had escalated its status to that of a global pandemic, signaling its far-reaching impact and the urgent need for international attention. Since then, the pandemic has proliferated globally, resulting in substantial morbidity and mortality [1-3]. Most COVID-19 patients experience low-to-moderate pneumonia and are fully recovered without medical treatment [4]. The range of clinical symptoms can be changeable from completely asymptomatic to severe pneumonia and death. As this is a newly emerged disease, while treatments have been developed and authorized for emergency use, the mainstay of health system performance remains focused on prevention and interrupting the transmission chain through vaccination, alongside providing supportive therapies for patients [5]. Although respiratory and general care details have been detected and improved, the mortality rate attributed to this disease is considerable. Some patients may need a mechanical ventilator and are hospitalized in intensive care units (ICU), which aggravates the prognosis of the disease. Hence, early detection of infection is essential to hamper disease progression [6].

Considering the risks that COVID-19 poses to the individual, which is seen only in a small fraction of people with severe COVID-19, it warrants having a reliable predictive method for COVID-19 severity to distinguish that susceptible minority from the majority with benign disease course and to protect them against critical consequences. This risk stratification, which forecasts the patient’s condition and disease progression, assists healthcare front liners in providing more tailored medical management and earlier therapeutic intervention for high-risk patients.

This study introduces a novel scoring system, amalgamating chest CT scan findings with demographic and clinical variables, aimed at predicting COVID-19 critical outcomes in patients. By correlating an encompassing severity score with pivotal clinical findings, we aspire to provide clinicians with a tool to promptly assess disease severity and manage patient care and resources efficiently, potentially shaping adaptive strategies in the face of an evolving healthcare landscape.

## Materials and methods

Ethical approval, data collection, and image acquisition

The Ethics Committee of Iran University of Medical Science (IUMS) approved this retrospective cohort study (IR.IUMS.FMD.REC.1400.467). Data were collected from 425 medical records from COVID-19-confirmed cases referred to Hazrate Rasool-e-Akram and Firoozabadi hospitals affiliated to IUMS, between February 2020 and April 2020. Four trained medical researchers evaluated data using a checklist containing demographic, clinical, and radiologic characteristics. Clinical and demographic variables were obtained from clinical records, and in case of possible missing data, follow-up phone calls were scheduled.

Chest CT scans were performed utilizing a standard protocol across two medical facilities to maintain consistency in the data acquisition process. The CT images were obtained using a Siemens SOMATOM device (Siemens Medical Solutions USA, Inc., Malvern, PA), and all scans were executed with patients in a supine position and preferred during end inspiration without the administration of contrast media. The commonly used scan parameters were as follows: tube voltage of 120 kVp, automatic tube current modulation (30-70 mAs), pitch of 1.2, and slice thickness of 5 mm. Image reconstruction was performed with a standard kernel, and the resulting images were viewed at window settings optimized for lung parenchyma (window width: 1,500 HU; window level: -700 HU). To minimize variability, a single board-certified radiologist (E.Z.), blinded to the clinical details and outcomes, evaluated the scans and assigned severity scores based on the extent of lung involvement, adhering to the predefined scoring criteria. All digital image data were stored and analyzed using the picture archiving and communication system (PACS), ensuring a structured and standardized evaluation.

Inclusion and exclusion criteria

Inclusion criteria encompassed confirmed COVID-19 cases, validated either through chest CT scans and clinical symptoms or through RT-PCR, only including cases aged 18 years or older [7]. The exclusion criteria comprised patients with incomplete clinical records, unavailable chest CT scans, or comorbid lung diseases such as COPD, tuberculosis, and asbestosis. A specialized infectious disease expert performed the COVID-19 diagnosis, relying on chest CT scan patterns and either clinical symptoms or a positive RT-PCR test.

Development of scoring tools

Patients were assessed for COVID-19 severity according to three checklists, which were completed based on demographic factors in Table 1, clinical factors in Table 2, and chest CT scan findings as follows: a score was calculated based on the extent of lung involvement for each of the five lobes. The scoring criteria are as follows: Less than 5% involvement is given a score of 1; involvement between 5% and 25% is given a score of 2; involvement from 26% to 49% is scored as 3; involvement ranging from 50% to 75% receives a score of 4; and greater than 75% involvement is scored as 5. The overall CT severity score is obtained by summing the individual scores assigned to each lobe. This composite score can range from zero, indicating no involvement, to a maximum of 25, which would signify extensive lung involvement in all five lobes.

**Table 1:** Demographic data scoring criteria BMI: Body Mass Index

Factor	Score		
	0	1	2
Age (Year)	< 50	50-65	> 65
History of diabetes	No	Control with medication	Uncontrolled and complicated diabetes
Hypertension	No	Control with medication	Uncontrolled
Smoker	Lifetime none-smoker or ex-smoker with < 10 packs per year	Ex-smoker: ≥ 10 packs per year	Current smoker
Asthma	No	-	Yes
BMI (kg/m^2^)	< 35	35-40	> 40
Immune system disorder	No	He has been undergoing chemotherapy for more than six months	under chemotherapy in the last six months or under recent treatment with immunosuppressive drugs
Liver or kidney failure	No	Kidney failure without dialysis	Dialysis or liver cirrhosis
Any mental disability	No	-	Yes
Any physical disability	No	-	Yes
Heart failure	No	-	Yes

**Table 2:** Clinical findings scoring criteria

Factor	Score		
	0	2	6
High fever (C)	T ≤ 38	38 < T ≤ 40	T > 40
Respiratory rate (RR) per minute	RR ≤ 24	24 < RR ≤ 30	RR > 30
Pulse rate (PR)	PR ≤ 100	101 < PR ≤ 120	PR > 120
Pulse Saturation of O_2_ (PSO_2_)	PSO_2_ ≥ 93%	90 < PSO_2_ < 93%	PSO_2_ < 90%
Respiratory distress	No	Relative use of secondary respiratory muscles	Suprasternal or intercostal retraction, clear use of respiratory accessory muscles

Subsequently, considering the disease progression, we stratified our patients into two outcome groups of severe COVID-19 and non-severe COVID-19 on the basis of occurrence of death, need for intubation, ICU admission, or prolonged hospital stay.

The radiologist estimated the CT severity score according to the American College of Radiology (ACR) criteria. The severity of the chest CT scan and its quantitative trend was reported by introducing a specific definition of CT score (which ranged from 0 to 25) and divided into four groups. Using this simple score, the range of lung involvement was classified by a radiologist from 0 (0% or nothing), 1 (1-5% or at least), 2 (6-25% or low), 3 (26-49% or moderate), 4 (50-75% or severe), and 5 (75% ≤ extended) [8]. We introduced a comprehensive severity score criterion, which comprised all three subscores of CT severity, demographic criteria, and clinical criteria and was assessed for its predictive value of COVID-19 disease severity. This compound score is composed of five levels, ranging from C1 to C5, as depicted in Table 3.

**Table 3:** Comprehensive severity score criteria composed of demographic, clinical, and radiologic sub-scores

Code	Reason for hospitalization
C1	Minimum overall score of 6 based on clinical findings
C2	A minimum overall score of 3 based on demographic characteristics and a minimum score of 4 based on clinical findings
C3	A minimum score of 4 based on clinical findings and CT severity score (10-15)
C4	A minimum overall score of 3 based on demographic characteristics and CT severity score (15-20)
C5	CT severity score (20-25)

The design of this decision-making tool is an innovative action and is not based on a specific reference. This comprehensive score was constructed through careful observation of the behaviors of experts, including emergency, lung, and infectious disease specialists, in making decisions in the field upon stratifying high-risk patients. Additionally, in line with the literature review, the weight of some disease symptoms in prognosis was obtained and was integrated as a criterion in this tool.

Here, a chest CT scan is performed at the physician's discretion and maintains a pivotal role in the evaluation for subsequent follow-ups. The decision for hospitalization is made based on the severity of chest CT scan results in C5 criteria. However, C3 and C4 criteria are in the gray zone, meaning the severity of clinical or chest CT scan results is not merely enough to decide on hospitalization. Hence, we postulated that, by a combination of these three different criteria, a predictive measure for hospitalization could be generated in which higher scores denote higher severity of the disease.

Statistical analysis

Descriptive analyses were applied for demographic, clinical, and chest CT severity score data. An Independent T-test was used to compare means in each group. Pearson's correlation and chi-square test were used for assessing categorical variables. Logistic regression was used for assessing independent predictor variables and controlling confounders. The receiver operating characteristic (ROC) curve was applied to estimate the chest CT scan severity cutoff score in predicting the outcomes in patients. P value < 0.05 was considered statistically significant. All analyses were performed using Statistical Product and Service Solutions (SPSS) (version 24; IBM SPSS Statistics for Windows, Armonk, NY).

## Results

Our studied population included 406 patients, comprising 172 (42.3%) women and 234 (57.7%) men. The mean ± SD age of patients was 61.6 ± 17.2 and ranged from 18 to 98. The mean ± SD of BMI was 25.9 ± 7.9. Considering management settings, 366 (90.1%) required hospitalization, and the other 40 (9.9%) were treated as outpatient. Finally, 161 (39.7%) were stratified as the "severe COVID-19" group, and 245 (60.3%) were in the "non-severe COVID-19" group.

The result of univariate analyses revealed that hospitalized condition, positive PCR test, older age, the median of nadir O_2_ saturation, psychological and physical disorders, cardiac disorder, liver cirrhosis, active cancer, and having chemotherapy in the last six months were significantly associated with severe outcome (p < 0.05). There are no significant differences in other demographic and clinical variables between the two groups (p > 0.05) (Table 4).

**Table 4:** Comparison of the demographic and clinical findings in two groups Statistics are mean ± standard deviation (SD), or number (%). P-value < 0.05 was considered statistically significant. PCR: Polymerase Chain Reaction, BMI: Body Mass Index, %SpO2: Percent Saturation of Peripheral Oxygen, HTN: Hypertension, COPD: Chronic Obstructive Pulmonary Disease, ACE Hx: History of Angiotensin-Converting Enzyme inhibitor use, ARB Hx: History of Angiotensin II Receptor Blocker use, N: Number.

Variable		Group		P-value
		Severe COVID-19 (N=161)	Non-severe COVID-19 (N=245)	
Sex	Male, N(%)	99 (61.5%)	135 (55.1%)	0.21
	Female, N(%)	62 (38.5%)	110 (44.9%)	
PCR (Positive), N(%)		32 (19.9%)	29 (11.8%)	0.043
Age (Years), Mean ± SD		68 ± 15	57 ± 17	0.001
BMI (Kg/m^2^), Mean ± SD		26 ± 9	26 ±7	0.085
Days between first related symptom and first visit, Mean ± SD		7 ± 9	6 ± 7	0.68
Diabetes (Yes), N(%)		55 (34.2%)	75 (30.6%)	0.45
HTN (Positive), N(%)		67 (41.6%)	80 (32.7%)	0.066
Obesity (Yes), N(%)		33 (20.5%)	68 (27.8%)	0.17
%SpO2 at the initial examination, Mean ± SD		77 ± 25	84 ± 24	0.002
Renal Insufficiency (Yes), N(%)		19 (12.8%)	16 (7.3%)	0.19
Disabling physical illness (Yes), N(%)		17 (10.6%)	3 (1.2%)	0.001
Disabling mental illness (Yes), N(%)		10 (11.8%)	3 (1.2%)	0.001
Heart Failure (Yes), N(%)		35 (21.7%)	32 (13.1%)	0.021
Cirrhosis (Yes), N(%)		6 (3.7%)	1 (0.4%)	0.012
COPD & Asthma (Yes), N(%)		19 (11.8%)	19 (7.8%)	0.17
Fever (Yes), N(%)		46 (28.6%)	82 (33.6%)	0.39
ACE Hx (Yes), N(%)		13 (8.1%)	12 (4.9%)	0.19
ARB Hx (Yes), N(%)		46 (28.6%)	51 (20.8%)	0.066
Metformin (Yes), N(%)		29 (18%)	51 (20.8%)	0.48
Tobacco current consumption (Yes), N(%)		36 (22.4%)	45 (18.37%)	0.098
Immunocompromised (Yes), N(%)		3 (1.9%)	2 (0.8%)	0.35
Chemotherapy last 6 months (Yes), N(%)		5 (3.1%)	1 (0.8%)	0.028
Active cancer (Yes), N(%)		8 (5%)	3 (1.2%)	0.023
Splenectomy (Yes), N(%)		1 (0.6%)	3 (1.2%)	0.028

The association of outcomes with demographic and clinical characteristics scores

A comparison of the median overall score of demographic and clinical characteristics indicated that the mean overall score of demographic factors was significantly higher for people in the severe COVID-19 group than that of the non-severe group (3.9 ± 3.32 vs 2.78 ± 2.15, respectively, p = 0.001). Moreover, the mean overall score of clinical criteria was significantly higher in the severe COVID-19 group than in the non-severe group (17.21 ± 8.86 vs 11.17 ± 9.93, respectively, p = 0.001). A comparison of chest CT scan outcomes revealed that the number of people with > 50% lung involvement was significantly higher in the severe COVID-19 group as 39 (24.3%) vs 14 (5.7%), respectively (p = 0.001). The distribution of different degrees of lung involvement between the two groups is depicted in Table 5.

**Table 5:** Comparison of frequency distribution of lung involvement findings in the two groups Statistics are numbers (%). P-value < 0.05 was considered statistically significant.

Lung involvement	Group		P-value
	Severe COVID-19 (N=161)	Non-severe COVID-19 (N=245)	
0 or none	3 (1.9%)	12 (4.9%)	0.001
1–5% or minimal	17 (10.6%)	67 (27.3%)	
6–25% or mild	61 (37.9%)	107 (43.7%)	
26–49% or moderate	41 (25.5%)	45 (18.4%)	
50–74% or severe	36 (22.4%)	14 (5.7%)	
≥ 75% or extensive	3 (1.9%)	0%	

According to the comprehensive CT score, the number of people that were categorized as the severe COVID-19 group was significantly higher in each of C1-C5 sub-groups (all p-values < 0.05) (Table 6).

**Table 6:** Comparison of outcome frequency based on comprehensive severity score criteria Statistics are numbers (%). P-value < 0.05 was considered statistically significant.

Inpatients index	Non-severe COVID-19 (N=245)	Severe COVID-19 (N=161)	P-value
C1 (yes)	147 (60%)	138 (85.7%)	0.001
C2 (yes)	123 (50.2%)	117 (72.7%)	0.001
C3 (yes)	115 (46.9%)	101 (62.7%)	0.001
C4 (yes)	7 (2.9%)	28 (17.4%)	0.001
C5 (yes)	0 (0%)	3 (1.9%)	0.001

The results of multivariate logistic regression analyses showed that hospital stay duration was predicted with C1, which was statistically significant. In other words, the odds of prolonged hospital stay duration were significantly higher in people with C1 level than the others (OR = 1.17, p = 0.001), and there was no significant association between C1 and the other outcomes. Being in C2 was anticipated intubation, being in C3 was projected with prolonged hospitalization and high odds of death (p > 0.05), and being in C4 was expected with the odds of intubation and death while having no significant association with the other outcomes (p > 0.05). Additionally, being in C5 predicted death, intubation, and ICU admission, and death with OR=2.25 was the most important predicting factor at this level (Table 7).

**Table 7:** Examining predictive outcomes based on CT comprehensive scores P-value < 0.05 was considered statistically significant. OR: Odds Ratio, CI: Confidence Interval

CT Comprehensive Score	Outcome	OR adjusted	95% CI	P-value
C1	Death	1.03	0.41-2.98	0.33
	ICU admission	1.01	0.36-3.65	0.89
	Intubation	1.09	0.49-1.11	0.086
	Hospital stay duration	1.17	1.09-1.35	0.001
C2	Death	1.01	0.46-2.64	0.25
	ICU admission	1.44	0.42-4.95	0.55
	Intubation	2.11	1.41-4.32	0.001
	Hospital stay duration	1.018	0.97-2.33	0.46
C3	Death	1.09	1.012-2.82	0.035
	ICU admission	1.3	0.43-4.33	0.64
	Intubation	1.64	0.85-3.1	0.13
	Hospital stay duration	1.12	1.005-1.19	0.03
C4	Death	1.44	1.18-2.85	0.001
	ICU admission	1.03	0.48-2.37	0.48
	Intubation	2.33	1.26-4.88	0.011
	Hospital stay duration	0.99	0.92-1.064	0.78
C5	Death	2.25	1.12-4.1	0.001
	ICU admission	2.11	1.03-4.9	0.002
	Intubation	1.98	1.33-4.38	0.042
	Hospital stay duration	1.04	0.818-1.23	0.77

Predicting factors for disease severity

According to multivariate regression analysis results, an increase in demographic (OR = 1.24, 95% CI = 1.125-1.372), clinical (OR = 1.028, 95% CI = 1.001-1.055), and chest CT scan (OR = 1.12, 95% CI = 1.066-1.190) scores was correlated with higher odds of death (p < 0.05 for all three scores).

**Figure 1:**
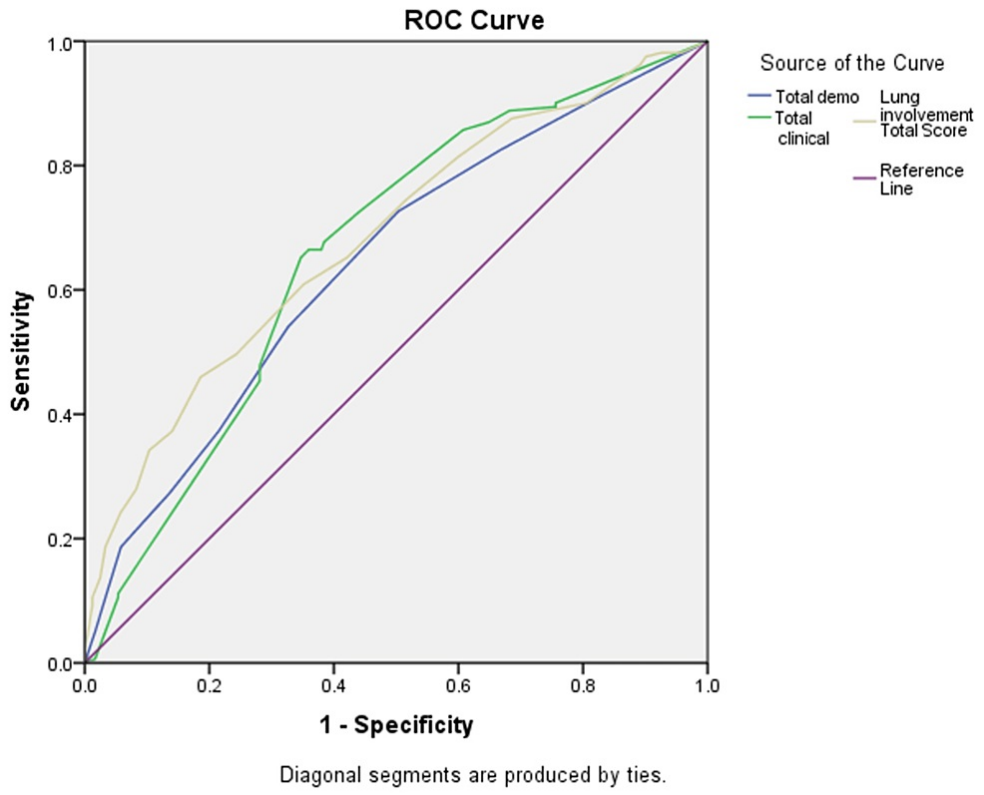
Determination of outcome severity prediction based on their demographic, clinical characteristics, and CT scan findings

Based on ROC curve analysis results (Figure 1), all three scores had satisfactory accuracy in predicting disease severity considering their > 50% area under the curve (AUC). The optimum cutoff value was calculated as 2.5 for the demographic score (sensitivity of 73%, specificity of 56%, AUC of 64%), 9.5 for the clinical score (sensitivity of 89%, specificity of 69%, AUC of 72%), and 8.5 for the CT severity score (sensitivity of 84%, specificity of 69%, AUC of 68%).

## Discussion

From the earliest emergence of SARS-CoV-2 infection in Wuhan, China, multiple severe forms of the disease have been delineated [3,9-11]. As the situation exacerbated and was declared a pandemic in March 2020, a worldwide effort was exerted by clinical researchers to identify the potential factors for disease severity and to forecast mortality and morbidity in different populations [6,12]. Some prognostic tools have been developed with diverse scoring methods, yet their assessment has been restricted to Western societies, leading to relatively scarce data in the Asian population [6].

This study revealed that the chest CT scan severity score and the proposed clinical and demographic scales served as reliable predictors for COVID-19 severity. The highness of the mentioned scores compared to the cutoff determined for them forecast a higher chance of death, hospitalization, ventilator support, and ICU admission. The odds of long hospitalization were significantly higher for people at the C1 level than the others. Being at the C2 level was predicted with intubation. Being in C3 was anticipated with death and prolonged hospitalization. C4 level foretold intubation and a higher probability of death. At the C5 level, the probability of death, intubation, and ICU admission was increased. 

In a study by Aziz-Ahari et al. [13] on a similar population, the degree of the chest CT scan involvement score in evaluating the severity of disease and short-term prognosis in 148 COVID-19 patients was assessed. Of these, 93 (62.8%) were discharged, and 55 (37%) deceased. The reported mortality rate of 55 patients (37%) was concordant with the percentage of our cases with severe outcomes 161 (39.7%). The study revealed that the extent of chest CT scan findings and employment of numeral scaling of lung involvement, in particular, could be practical in predicting severe outcomes, aligning with our findings.

Wang et al. [14] conducted a retrospective cohort study on 239 COVID-19 patients, aiming to introduce a new scoring method for predicting disease development from low to severe. A majority, 216 (90.38%), were in the mild/moderate group, and 23 (9.62%) were in the severe group. The average lung involvement rate in our study was 39.6%, which was higher than this study, possibly due to differences in population characteristics, immunity of patients, health services accessibility, and sample size. They proposed a new score (PAINT), based on lung disease, age >75 years old, immunoglobulin M, CD16+/CD56+ natural killer cells, and aspartate aminotransferase, demonstrating high predictive value.

Li et al. [15] conducted a study that evaluated clinical and severe and critical chest CT scan factors of COVID-19 patients, including 83 patients in total. Of these, 25 (30.1%) were severe cases, and 58 (69.9%) were non-severe. The results showed that the sensitivity and specificity of chest CT scans for distinguishing these two were 80% and 82.2%, respectively, which were slightly lower than those of our results. Given the smaller sample size of this study compared to ours, this difference can be justified. Clinical factors, such as age > 50 years old, comorbidity, apnea, chest pain, cough, decrease in lymphocytes, and increased inflammation measures, were identified as risk factors for the development of the severe form of COVID-19, which is consistent with our study. Generally, this study revealed significant differences in clinical symptoms, laboratory results, and chest CT scans between non-severe and severe/critical patients and ascertained the reliability of chest CT scan involvement in predicting outcomes in patients.

The major drawback to previous studies is the limitation of factors considered in predictive criteria. By the inclusion of multiple factors and proposing three distinct criteria, we were able to assess the weight of similar factors combined together and independent from other criteria. This allows us to take adept preventive measures while making policies for disease prevention and to mitigate mortality and morbidity of this infection. Importantly, the most unique feature of this study is conferring numeric scoring to the proposed multi-disciplinary criteria. With the implementation of the confirmed cutoff points of scoring scales, we could carry out quantitative risk stratification for patients at the initial visit to the emergency department and prioritize healthcare resources for cases at higher risk to improve overall prognosis. This is highly crucial, particularly in periods of disease surge, which engenders catastrophic shortages of health service resources, a phenomenon observed recurrently in the COVID-19 pandemic.

In this study, a more comprehensive scoring method based on the first chest CT scan, demographic factors comprising old age, smoking, comorbidity like diabetes, clinical characteristics, and chest CT scan results such as the severity of lung involvement, has been proposed, which was proven to maintain high value in predicting several outcomes (death, hospitalization, intubation, and ICU admission), and its predicting power was confirmed by ROC curve. Hence, we constructed an exhaustive forecast method so that no study has been carried out with such magnitude.

Limitations and strengths of the study

Our study presents both notable strengths and limitations that warrant discussion. A primary limitation lies in its retrospective design, which inherently faces challenges, such as incomplete records. Such gaps in data could potentially impact the study's outcomes, suggesting that a prospective approach would yield more accurate results. Conversely, a significant strength of our research is the implementation of a comprehensive scoring system that integrates a wide array of factors - demographic, clinical, and radiographic - to predict patient outcomes, enhancing the study's robustness and applicability. Additionally, the validity of our prognostic tool may vary in subsequent pandemic peaks due to evolving viral strains and clinical practices, necessitating further validation during diverse pandemic phases.

Even though the radiologist estimated the CT severity score based on defined criteria, we admit that radiologic interpretation inevitably incorporates an element of subjectivity, which could introduce variability into the results. Different radiologists may interpret images with subtle differences, and while our scoring system provides a structured framework, inherent subjectivity may still impact scoring, especially in borderline cases. This potential for variability and its subsequent influence on the study outcomes warrant acknowledgment as a limitation. One way to overcome such limitations is the incorporation of artificial intelligence-based image analysis to mitigate such subjectivity, ensuring even greater consistency and reliability in data interpretation.

## Conclusions

Our study indicated that the total number of demographic, clinical, and radiologic characteristics are independently related to severe forms of COVID-19. The scoring method based on the first chest CT scan (SMBF-CT) scale, which is calculated based on demographic, clinical, and radiographic findings of patients, was proven to predict the outcomes of COVID-19 patients and is useful for treatment decision-making. Thus, utilizing SMBF-CT can effectively detect disease earlier and decrease the severe outcomes and burden of disease.
